# Implementing Palliative Care Consultation Programs in Nursing Homes: Key Stakeholders’ Perspectives

**DOI:** 10.1093/geroni/igaf122.4138

**Published:** 2025-12-31

**Authors:** Elisha Oduro, Alexandra Mora, Ruth Lopez, Kathleen Unroe, Moses Akwobugi, Joan Carpenter

**Affiliations:** University of Maryland Baltimore, Baltimore, Maryland, United States; University of Maryland Baltimore, Baltimore, Maryland, United States; MGH Institute of Health Professions, Sharon, Massachusetts, United States; Indiana University School of Medicine, Indiana, Indiana, United States; University of Maryland Baltimore, Baltimore, Maryland, United States; University of Maryland School of Nursing, Baltimore, Maryland, United States

## Abstract

Palliative care (PC) consultation is associated with reduced acute care use and burdensome transitions for nursing home (NH) residents. However, little is known about the steps for implementing PC consultations into NHs. This study identified the processes of implementing PC consultations into NH workflows from the perspective of key stakeholders. Using a qualitative descriptive approach, we conducted semi-structured interviews with stakeholders in fourteen NHs in the Eastern United States. We analyzed transcribed interviews using conventional content analysis. Participants (N = 33) were NH direct care workers (e.g., nurses, social workers), PC consultants (e.g., PC nurse practitioners), and leaders (e.g., PC agency and NH directors), predominantly female (93.9%), and White (93.9%). One-third had 20 years of experience working in NHs. We identified a six-step PC consultation implementation process: (1) NH and PC agency establishing partnerships, (2) NH staff identifying and determining the need for PC referral, (3) NH staff initiating PC referrals, (4) PC agency processing referrals, (5) PC provider conducting consult, and (6) PC and NH follow-up to ensure PC care is implemented. Within each step, NH residents and their families, PC agency and NH leaders, and primary care providers significantly shape the process by undertaking actions that either move or stall the PC consultation process. Strategies that support the process include strong NH and PC agency relationships, PC consultant provider availability, PC consultant access to resident information, and NH staff investment in PC consultation services. Our findings provide an actionable framework to guide the implementation of effective NH PC consultations.

